# Applications of Nanotechnology in Plant Growth and Crop Protection: A Review

**DOI:** 10.3390/molecules24142558

**Published:** 2019-07-13

**Authors:** Yifen Shang, Md. Kamrul Hasan, Golam Jalal Ahammed, Mengqi Li, Hanqin Yin, Jie Zhou

**Affiliations:** 1 Department of Horticulture, Zijingang Campus, Zhejiang University, Yuhangtang Road 866, Hangzhou 310058, China; 2 Department of Agricultural Chemistry, Sylhet Agricultural University, Sylhet 3100, Bangladesh; 3 College of Forestry, Henan University of Science and Technology, Luoyang 471023, China; 4 Zhejiang Institute of Geological Survey, Xiaojin Road 508, Hangzhou 311203, China

**Keywords:** nanotechnology, nanoagrochemicals, nanosensors, nanobionics, sustainable agriculture, food security

## Abstract

In the era of climate change, global agricultural systems are facing numerous, unprecedented challenges. In order to achieve food security, advanced nano-engineering is a handy tool for boosting crop production and assuring sustainability. Nanotechnology helps to improve agricultural production by increasing the efficiency of inputs and minimizing relevant losses. Nanomaterials offer a wider specific surface area to fertilizers and pesticides. In addition, nanomaterials as unique carriers of agrochemicals facilitate the site-targeted controlled delivery of nutrients with increased crop protection. Due to their direct and intended applications in the precise management and control of inputs (fertilizers, pesticides, herbicides), nanotools, such as nanobiosensors, support the development of high-tech agricultural farms. The integration of biology and nanotechnology into nonosensors has greatly increased their potential to sense and identify the environmental conditions or impairments. In this review, we summarize recent attempts at innovative uses of nanotechnologies in agriculture that may help to meet the rising demand for food and environmental sustainability.

## 1. Introduction

To address the increasing challenges of sustainable production and food security, significant technological advancements and innovations have been made in recent years in the field of agriculture [1,2,3]. Such continuous agricultural innovations are crucial to meet the increasing food demand of exploding global population through the uses of natural and synthetic resources. In particular, nanotechnology has potential to provide effective solutions to the multiple agriculture-related problems. To bridge the gap between bulk materials and atomic or molecular structures, nanoparticles provide a great scientific interest. Over the last two decades, a significant amount of research has been carried out on nanotechnology emphasizing its numerous applications in agriculture sectors [4,5,6]. Fertilizer application plays a pivotal role in increasing the agricultural production; however, the excessive usages of fertilizers irreversibly alter the chemical ecology of soil, further reducing the available area for crop production. Sustainable agriculture entails a minimum use of agrochemicals that can eventually protect the environment and conserve different species from extinction. Notably, nanomaterials enhance the productivity of crops by increasing the efficiency of agricultural inputs to facilitate site-targeted controlled delivery of nutrients, thereby ensuring the minimal use of agri-inputs. Indeed, the assistance of nanotechnology in plant protection products has exponentially increased, which may assure increased crop yield. Moreover, the major concern in agricultural production is to enable accelerated adaptation of plants to progressive climate change factors, such as extreme temperatures, water deficiency, salinity, alkalinity and environmental pollution with toxic metals without threatening existing sensitive ecosystems [7]. In addition, the development and exploitation of nanosensors in precision farming, to measure and monitor crop growth, soil conditions, diseases, uses and penetration of agrochemicals and environmental pollution have substantially improved the human control of soil and plant heath, quality control and safety assurance contributing much to sustainable agriculture and environmental systems [5]. Nanomaterial engineering is the cutting-edge track of research that supports the development of high-tech agricultural fields by offering a wider specific surface area crucial for the sustainable development of agriculture systems [8,9]. Therefore, nanotechnology can not only reduce the uncertainty, but also coordinate the management strategies of agricultural production as an alternative to conventional technologies. In many instances, agro-nanotech innovations offer short-term techno-fixes to the problems faced in modern industrial agriculture. The present review summarizes the applications of nanotechnology in agriculture, which may ensure the sustainability of agriculture and environment.

## 2. Nano-Farming: A New Frontier in Agricultural Development

**Figure 1:**
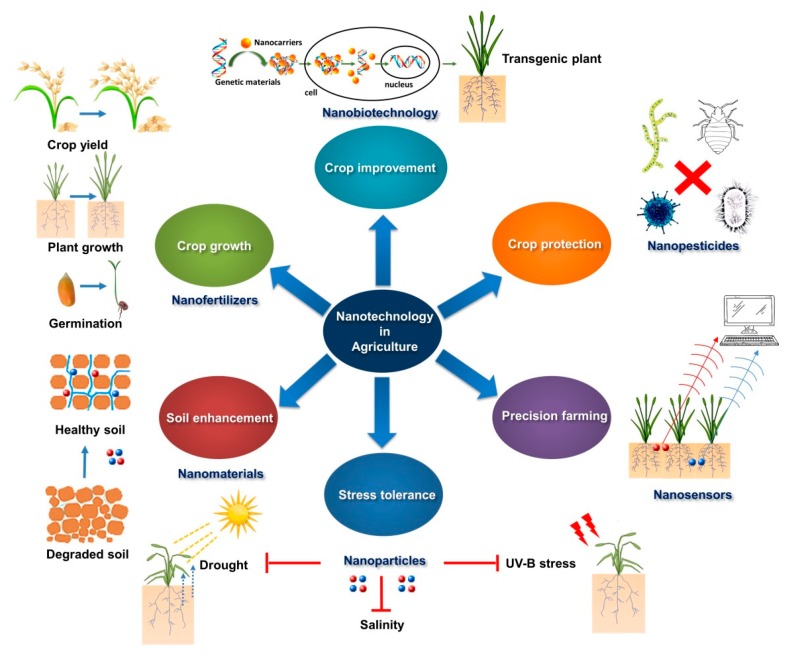
Applications of nanotechnology in agriculture. Controlled released nanofertilizers improve crop growth, yield and productivity. Nano-based target delivery approach (gene transfer) is used for crop improvement. Nanopesticides can be used for efficient crop protection. Uses of nanosensors and computerized controls greatly contribute to precision farming. Nanomaterials can also be used to promote plant stress tolerance and soil enhancement. Readers are referred to the text for further details. (Modified and redrawn from references [17,18,19,20,21,22,23]).

Nanoparticle engineering is one of the latest technological innovations that demonstrate unique targeted characteristics with elevated strength. The term ‘nanotechnology’ was first coined by Norio Taniguichi, a professor at Tokyo University of Science, in 1974 [10]. Although, the term ‘nanotechnology’ has long been introduced in multiple disciplines, the idea that nanoparticles (NPs) could be of interest in agricultural development is a recent technological innovation, and is still under progressive development [11]. Recent advancements in the fabrication of nanomaterials of different sizes and shapes have yielded their wide array of applications in medicine, environmental science, agriculture and food processing. Throughout history, agriculture has always benefited from these innovations [5]. In continuation, as agriculture faces numerous and unprecedented challenges, such as reduced crop yield due to biotic and abiotic stresses, including nutrient deficiency and environmental pollution, the emergence of nanotechnology has offered promising applications for precision agriculture (Figure 1). The term precision agriculture or farming has emerged in recent years, meaning the development of wireless networking and miniaturization of the sensors for monitoring, assessing, and controlling agricultural practices. More specifically, it is related to the site-specific crop management with a wide array of pre- and post-production aspects of agriculture, ranging from horticultural crops to field crops [1]. Recent advancements in tissue engineering and engineered nanomaterials-based targeted delivery of CRISPR (clustered regularly interspaced short palindromic repeats)/Cas (CRISPR-associated protein) mRNA, and sgRNA for the genetic modification (GM) of crops is a noteworthy scientific achievement [12,13,14]. Further, nanotechnology provides excellent solutions for an increasing number of environmental challenges. For example, the development of nanosensors has extensive prospects for the observation of environmental stress and enhancing the combating potentials of plants against diseases [15,16]. Therefore, such continuous improvements in nanotechnology with special preference on the identification of problems and development of collaborative approaches for sustainable agricultural growth has remarkable potential to provide broad social and equitable benefits.

## 3. Sources and Synthesis of Green Nanoparticles

Nanoparticles (NPs) are organic, inorganic or hybrid materials with at least one of their dimensions ranging from 1 to 100 nm (at the nanoscale). NPs that exist in the natural world can be produced from the processes of photochemical reactions, volcanic eruptions, forest fires, simple erosion, plants and animals or even by the microorganisms [24,25]. The production of plant- and microorganism- derived NPs, has emerged as an efficient biological source of green NPs that draw an extra attention of scientist in recent times due to their eco-friendly nature and simplicity of production process compared to the other routes [8,25,26,27,28]. For the exploitation of the green nanotechnology, a number of plant species and microorganisms including bacteria, algae and fungi are being currently used for NP synthesis. For example, *Medicago sativa* and *Sesbania* plant species are used to formulate gold nanoparticles. Likewise, inorganic nanomaterials, made of silver, nickel, cobalt, zinc and copper, can be synthesized inside live plants, such as *Brassica juncea, Medicago sativa and Heleanthus annus* [8,20,26,27]. Microorganisms, such as diatoms, *Pseudomonas stuzeri*, *Desulfovibrio desulfuricans* NCIMB 8307 *Clostridium thermoaceticum* and *Klebsiella aerogens* are used to synthesize silicon, gold, zinc sulphide and cadmium sulphide nanoparticles, respectively. Although a large number of microorganisms are used to synthesize green NPs, fungi, mainly *Verticillium* sp., *Aspergillus flavus*, *Aspergillus furnigatus*, *Phanerochaete chrysoparium* and *Fusarium oxysporum* are considered to be the most efficient systems for the biosynthesis of metal and metal sulphide containing NPs [8,26].

All NPs are three-dimensional (3D) objects. One dimensional (1D) NPs refers to the NPs that have 2 dimensions at the nanoscale and 1 dimension at the macroscale (nanowires, nanotubes), whereas two-dimensional (2D) NPs have 1 dimension at the nanoscale and 2 dimensions at the macroscale (nanolayers, nanofilms). Again, 3D NPs have 0 dimension at the nanoscale and 3 dimensions at the macroscale (nanoballs, nanoflowers), whereas zero-dimensional (0D) NPs are characterized by all 3 dimensions at the nanoscale [29,30]. For instance, a rich variety of physical and chemical methods have been developed in favor of synthesis or fabrication of the zero-dimensional NPs with well-controlled dimensions [30]. Zero-dimensional NPs, such as quantum dots have wide acceptability and application in light emitting diodes, solar cells, single-electron transistors as used in lasers [31,32,33]. The synthesis of two-dimensional NPs, such as junctions (continuous islands), branched structures, nanoprisms, nanoplates, nanosheets, nanowalls, and nanodisks have become a crucial area in nano-engineering research [30]. Such geometric structures of NPs have blown up the investigation and development of novel applications in sensors, photocatalysts, nanocontainers and nanoreactors [34]. In contrast, three-dimensional NPs have recently attracted considerable research interest owing to their large surface area and other superior properties like absorption sites for all involved molecules in a small space which lead to a better transport of the molecules [30,35]. Therefore, the improvement and development of novel technology for the production of NPs with their potential application have special significance, particularly in the development of sustainable agricultural and environmental systems [36]. 

## 4. Nanoparticle-Enabled Smart Delivery Options: A New Window for Sustainable Agriculture

Nanotechnology is considered as one of the key technologies in the twenty-first century that promises to advance traditional agricultural practices and offer sustainable development by improving the management and conservation tactics with reduced waste of agricultural inputs [37,38]. The delivery systems of agrochemicals and organic molecules including transport of DNA molecules or oligonucleotides into the plant cells are important aspects of sustainable agricultural production as well as precision farming [39]. In conventional methods, agrochemicals are generally applied to crops by spraying and/or broadcasting. As a result, a very low number of agrochemicals reaches the target sites of crops, which is much below the minimum effective concentration required for successful plant growth. The losses are due to leaching of chemicals, degradation by photolysis, hydrolysis and also by microbial degradation [40,41]. For instance, in the case of the application of fertilizer, more emphasis should be made of the bioavailability of nutrients due to the chelation effects of soil, degradation by microorganisms, evaporation, over-application, hydrolysis, and run-off problems [11]. In the case of pesticide applications, the efficacy enhancement with spray drift management is to be focused on [20]. In order to ensure eco-friendly agricultural practices, the recent advancement of nanotechnology-based synthesis of slow or controlled release fertilizers, pesticides and herbicides has, therefore, received an extra attention in agriculture farming [8]. With the passage of time, nanotechnology has gradually moved from the lab-based experimental trials to practical applications. The aim of controlled delivery techniques is to release measured amount of necessary and sufficient quantities of agrochemicals over a period of time and to obtain the full biological competency with minimizing the loss and harmful effects [42]. Nanoparticles offer the advantages of effective delivery of agrochemical due to their large surface area, easy attachment and fast mass transfer [20]. For these reasons, micronic or submicronic particles are incorporated into the agrochemicals through several mechanisms, such as capsulation, absorption, surface ionic or weak bond attachments and entrapment into the nano-matrix of active ingredients [8,43,44]. For example, the capsulation of potassium nitrate by graphene oxide films considerably prolongs the fertilizer release process and such a formulation seems to be possible at a relatively low cost under large scale production [45]. Nanomaterials improve stability of agrochemicals and protect them from degradation and subsequent release into the environment, which eventually increase the effectiveness and reduce the quantities of agrochemicals.

**Figure 2:**
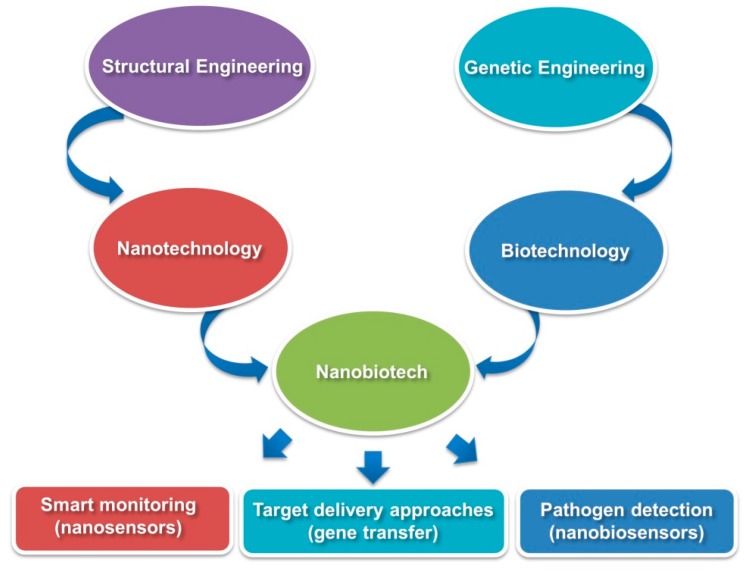
An overview of nanobiotechnology. Convergence of nanotechnology and biotechnology results in nanobiotech, which entails knowledge of structural engineering and genetic engineering. Nanobiotechnologies are used for different purposes in agriculture, including smart monitoring (nanosensors), target delivery of nucleic acid (gene transfer) and plant pathogen detection (nanodiagnostics). (Modified and redrawn from references [20,21,23]).

Apart from the agricultural applications, the convergence of nanotechnology with biotechnology also provides opportunities as new tools of molecular transporter to modify genes and even produce new organisms (Figure 2) [46]. For instance, nanobiotechnologies implicate nanoparticles, nanocapsules, and nanofibres to carry foreign DNA and the chemicals that facilitate to modify the target genes. During the delivery of genetic materials, viral gene delivery vectors face numerous challenges, such as limited host range, limited size of inserted genetic material, transportation across the cell membrane and also trafficking problem of the nucleus [20]. In contrast, the recent breakthroughs in nanobiotechnology provide greater opportunities to the researchers to completely replace the genetic material of one species by another [47]. In genetic engineering, silicon dioxide nanoparticles have been devised to deliver DNA fragments/sequences to the target species, such as tobacco and corn plants without any undesirable side effects [36,48]. In addition, NP-assisted delivery system is also used to develop insect resistant novel crop varieties. For examples, DNA-coated NPs are used as bullets in gene-gun technology for bombardment of cells or tissues to transfer the desired genes to the target plants [46,49]. The recent progress in chitosan NPs entrapped SiRNA delivery vehicles has provided a new plot of crop improvement allowing the target specific control of insect pests as chitosan has an efficient binding potential with RNA as well as penetration ability through the cell membranes [50]. Contemporary advances in nanomaterials-based specific delivery of CRISPR/Cas9 single guide RNA (sgRNA) has commenced a new era in genetic engineering. The CRISPR/Cas9 system comprising CRISPR repeat-spacer arrays and Cas proteins, is an RNA-directed defense system in prokaryotes and has successfully been used for genome editing in plants [13]. However, the low delivery efficiency is still a big hurdle impeding its applications. Interestingly, nanomaterials could minimize the degree of off-target changes by improving the efficiency and specificity of CRISPR/Cas systems. For example, cationic arginine gold nanoparticles (ArgNPs) assembled Cas9En (E-tag)-RNP (ribonucleoproteins) delivery of sgRNA provides about 30% effective cytoplasmic/nuclear gene editing efficiency in cultured cell lines, which would greatly facilitate future research into crop development [51].

**Table 1:** Effect of nanomaterials on crop physiology and plant protection.

Nanomaterials	Crop Species	Mode of Application	Concentrations Used	Duration of Treatments	Responses	References
MWCNTs	*Hordeum vulgare*, Glycine max, *Zea mays*	Seed priming	100 μg/mL	24 h	Enhanced germination and growth of seedlings	Lahiani et al. [52]
MWCNTs	*Triticum aestivum, Zea mays, Arachis hypogaea, Allium sativum*	Seed priming	50 μg/mL	Over night	Improved and rapid germination, increased biomass accumulation and water absorption potential of seeds	Srivastava and Rao [53]
ZnO	*Coffea arabica*	Foliar spray	10 mg/L	45 days	Enhanced growth, biomass accumulation and net photosynthesis	Rossi et al. [54]
ZnO	*Triticum aestivum*	Mixed with growth substrate	20 mg/L	Growth cycle	Increased grain yield and biomass accumulation	Du et al. [55]
ZnO	*Cyamopsis tetragonoloba*	Foliar spray	10 mg/L	6 weeks	Improved plant growth, biomass accumulation and nutrient content	Raliya et al. [56]
FeS_2_	*Cicer arietinum; pinacia oleracea; Daucus carota, Brassica juncea and Sesamum indicum*	Seed priming	80–100 μg/mL	12–14 h	Increased germination and crop yield	Srivastava et al. [57,58] Das et al. [59]
CuO	*Spinacia oleracea*	Mixed with soils	200 mg/kg	60 days	Improved photosynthesis and biomass production	Wang et al. [60]
ZnO	*Nicotiana tabacum*	Hydroponics	0.2 µM and 1 µM	21 days	Positively affected growth physiology, increased metabolites, enzymatic activities and anatomical properties of plants	Tirani et al. [61]
Fe/SiO_2_	*Arachis hypogaea, Zea mays*	As fertilizers	15 mg/kg	3 days	Enhanced plants growth and biomass accumulation	Disfani et al. [62]
TiO_2_	*Spinacia oleracea*	Seed priming and foliar application	0.25% suspension	48 h and 35 days	Increased biomass accumulation, chlorophyll, nitrogen and protein content.	Yang et al. [63]
AgNPs	*Triticum aestivum*	Mixed with pot soils	50 mg/L and 75 mg/L	Trifoliate stage	Improved growth and tolerance to heat stress	Iqbal et al. [64]
Ag NPs	*Vigna sinensis*	Foliar application	50 mg/L	40 days	Enhanced growth and biomass by stimulating root nodulation and soil bacterial diversity	Pallavi et al. [65]
TiO_2_ and SiO_2_	*Oryza sativa*	Foliar application	20 and 30 mg/L	55 days	Mitigated Cd toxicity and improved growth by stimulating antioxidant potential and inhibiting Cd translocation	Rizwan et al. [66]
SiO_2_ NPs	*Oryza sativa*	Foliar application	2.5 mM/L	70 days	Alleviated heavy metal toxicity and improved growth by decreasing bio-concentration and translocation in plants	Wang et al. [67]
ZnO, CuO and Ag NPs	*Prunus domestica* fruits	Fruit spray	100 and 1000 μg/mL	4 days	Suppressed grey mold symptoms caused by *B. cinerea* and soil borne diseases	Malandrakis et al. [68]
Al_2_O_3_ NPs	*Solanum lycopersicum*	Foliar application	400 mg/L	20 days	Successfully controlled *Fusarium* root rot in tomato	Shenashen et al. [69]
Ag NPs	*Vigna unguiculata*	Foliar application	50–100 μg/mL	7 Days	Showed no phytotoxicity, but could inhibit growth of *Xanthomonas axonopodis* pv. *malvacearum* and *Xanthomonas campestris* pv. *campestris* in vitro	Vanti et al. [70]
CuO	*Solanum lycopersicum*	Foliar application	150–340 μg/mL	11 days	Effectively controlled late blight disease caused by *Phytophthora infestans*	Giannousi et al. [71]
MgO	*Solanum lycopersicum*	Drenching	7–10 μg/mL	7 Days	Controlled bacterial wilt disease by suppressing pathogen *Ralstonia solanacearum*	Imada et al. [72]

## 5. Nanofertilizers: An Efficient Source of Balanced Crop Nutrition 

In general, the supplementation of the essential nutrients (element fertilization) is inevitable for improving crop productivity and soil fertility [73]. Nonetheless, the precise fertilizer management is considered as one of the most important prerequisites for sustainable agricultural development [74,75]. However, food is a fundamental human right. Global food security is under serious challenge across the world. Food security is threatened partly due to the limitation of available natural resources. It has been anticipated that current world population (seven billion) will increase over time and reach around nine billion by 2050. To feed the increasing population, about 60–100% more food will be needed [76]. To meet the increased food demand, intensive farming is being practiced which eventually leads to a vicious cycle of exhaustion of soil fertility and decline of agricultural yields. It has been estimated that approximately 40% of the world’s agricultural land has seriously been degraded, leading to a severe loss in soil fertility due to such intensive farming practices [77]. Consequently, a huge amount of fertilizers is used to improve soil fertility and crop productivity [38,78]. It has also been unequivocally observed that one third of crop productivity is attributed to fertilizers and the rest depends on use efficiencies of other agricultural inputs. Nonetheless, the nutrient use efficiencies of conventional fertilizers hardly exceed 30–40% [76]. For example, the nutrient use efficiency of conventional fertilizers, such as for nitrogen (N) 30–35%, phosphorus (P) 18–20%, and potassium (K) 35–40% remained constant for the past several decades [79]. Additionally, the nutrient use efficiency of conventional fertilizers that are applied directly into the soil, or sprayed on the leaves, largely depends on the final concentration of the fertilizers reaching the target sites [80]. In true sense, a very low amount, which is much below the minimum desired concentration, reaches the targeted site due to leaching loss of chemicals, drift, runoff, hydrolysis, evaporation, photolytic or even microbial degradation [81]. As consequences, the repeated use of excess amount of fertilizers adversely affects the inherent nutrient equilibrium of the soil. Beside these, water environments have seriously been contaminated due to leaching of toxic materials into rivers and water reservoirs, which also causes the contamination of drinking water [80]. It has been reported that in early 1970, only 27 kg NPK ha^−1^ was required to produce one ton of grain, whereas in 2008 it raised to 109 kg of NPK ha^−1^ to achieve the same level of production. According to the International Fertilizer Industry Association (IFIA), world fertilizer consumption has been increasing sharply and the world demand was projected to reach 192.8 Mt by the year 2016–2017 [82]. Among these large amounts of conventional fertilizers, a major portion of the chemicals remain in the soil or may enter into the other environmental compartments, resulting in severe environmental pollution that can affect the normal growth of flora and fauna.

The use of engineered nanomaterials in the frame of sustainable agriculture has shown a completely new way of food production that could potentially overcome the uncertainty in crop sector with limited available resources [83]. The revolution of green nanotechnology has dramatically changed the global agriculture canvass and nanomaterials as nanofertilizers have aroused promises to meet the projection of global food demand and sustainable agriculture as well. In order to alleviate the macro- and micro-nutrient deficiency through enhanced nutrient use efficiency and to overcome the chronic problem of eutrophication, nanofertilizers can be a best alternative [84]. Nanofertilizers synthesized in specific intension to regulate the release of nutrients depending on the requirements of the crops while minimizing differential losses, have immense potentiality. For example, conventional nitrogen fertilizers are manifested by huge losses from the soil through leaching, evaporation, or even the degradation of up to 50–70%, which ultimately reduces the efficiency of fertilizers and elevates the cost of production [41,85,86]. On the other hand, nanoformulations of nitrogenous fertilizers synchronize the release of fertilizer-N with their uptake demand by crops. Accordingly, nanoformulations prevent undesirable losses of nutrient via direct internalization by crops, and thereby avoiding the interaction of nutrients with soils, water, air and microorganisms [1,8]. For instance, the application of porous nanomaterials, such as zeolites, clay or chitosan significantly reduces the losses of nitrogen by regulating the demand-based release and enhancing the plant uptake process [8,87,88]. Ammonium charged zeolites have the potentiality to increase the solubility of phosphate minerals and thus exhibit improved phosphorus availability and uptake by crops [1]. Graphene oxide films, a carbon-based nanomaterial, can prolong the process of potassium nitrate release, which extends the time of function and minimizes losses by leaching [89]. Sabir et al. [81] also showed an excellent effort to open-up the potentiality of nanomaterials in crop production over traditional fertilizers. They demonstrated that nanocalcite (CaCO_3_-40%) application with nano SiO_2_ (4%), MgO (1%), and Fe_2_O_3_ (1%) not only improved the uptake of Ca, Mg, and Fe, but also notably enhanced the intake of P with micronutrients Zn and Mn. There are many different forms of nanofertilizers. Based on their actions, nanofertilizer could be classified as control or slow release fertilizers, control loss fertilizers, magnetic fertilizers or nanocomposite fertilizers as combined nanodevice to supply wide range of macro- and micro-nutrients in desirable properties [8,90]. Nanofertilizers are mainly produced by the encapsulation of nutrients with nanomaterials. Initial nanomaterials are produced by using both physical (top-down) and chemical (bottom-up) approaches, afterward the targeted nutrients are encapsulated inside nano porous materials or coated with thin polymer film or delivered as particles or emulsions of nanoscale dimension as it is for cationic nutrients (NH_4_^+^, K^+^, Ca^2+^, Mg^2+^) or after surface modification for anionic nutrients (NO_3_^−^, PO_4_^−^, SO_4_^−^) [8,79]. 

Agricultural production can be increased by 35–40% through balanced fertilizer management, irrigation and use of quality seeds. It has been keenly observed that application of nanoformulated fertilizers has significant potential to increase crop productivity. For example, the use of carbon nanoparticles together with fertilizer can increase grain yields of rice (10.29%), spring maize (10.93%), soybean (16.74%), winter wheat (28.81%) and vegetables (12.34–19.76%) [91]. Abdel-Aziz et al. [88] demonstrated that application of chitosan-NPK fertilizer significantly increases the harvest index, crop index and mobilization index of the determined wheat yield variables, as compared with control yield variables. Nanomaterials stimulate a number of vital facets of plant biology as plant root and leaf surfaces are the main nutrient gateway of plants which are highly porous at the nanoscale [92,93]. Consequently, the application of nanofertilizers can improve plant nutrient uptake through these pores, or the process can facilitate complexation with molecular transporters or root exudates through the creation of new pores, or by the exploitation of endocytosis or ion channels [94]. Moreover, it has been unequivocally observed from an extensive number of research that the size reduction of nanomaterials facilities the increase of surface mass ratio of particles, as a result, a bountiful amount of nutrient ions get adsorbed and desorbed slowly and steadily for an extended period of time [79,95]. Thus, the nanoformulations of fertilizers ensure a balanced nutrition of crops throughout the growth cycle that eventually improve agricultural production. It is to be noted that the increased efficiency of a product may encourage the farmers to use the product more profitably. 

As a promising interdisciplinary research field, nanotechnology has aroused its enormity in agriculture. In addition to macronutrients, micronutrients like manganese, boron, copper, iron, chlorine, molybdenum, zinc also play an integral role in steady increase of crop productivity. However, numerous factors, such as soil pH (alkalinity or acidic condition), stimulate their deficiencies in crop production with extensive farming practice [20]. The deficiency of micronutrients decreases not only the productivity of crops, but also affects human health through the consumption of micronutrient-deficient foods. For instance, iron deficiency causes anemia, growth impairment, reproductive health problems and even decreased cognitive and physical performance in humans [95,96]. In contrast, the supplementation of nanoformulated or nano entrapped micronutrients for the slow or controlled release of nutrients would stimulate the uptake process by plants, promote the growth and productivity of crops, and contribute to maintaining soil health as well [97]. For example, in zinc deficient soil, application of nano zinc oxide at low doses positively influences the growth and physiological responses, such as shoot and root elongation, the fresh dry weight and photosynthesis in many plant species compared to the control [98,99]. Kale et al. [77] also showed that application of zinc oxide nanoparticles with other fertilizer in zinc deficient soil, not only promotes nutrient use efficiency but also increases barley productivity by 91% compared to the control, whereas traditional bulk ZnSO_4_ increases productivity by 31% compared to the control. 

Scientific innovations are aimed to the betterment of human welfare. Likewise, plant scientists aim to restore the natural genomic diversity of different domesticated crops and to enhance technologies to reduce fertilizer consumption without compromising crop productivity and sustainable environment [59]. In procession, a new term, ‘control loss fertilizer’, is being used in sustainable agriculture. Such kind of fertilizers have been engineered to reduce the non-point pollution of inputs in agriculture that function by forming a nano network through self-assembly upon contact with water in soil [100]. Whereas, the entrapped fertilizer nutrients enter into the soil network via hydrogen bonds, surface tension, molecular force or viscous force. Consequently, their spatial scale enlarges so that they are easily blocked by soil filtration and remain fixed in soil around the crop roots, which facilitates nutrient absorption by plants to meet the demands throughout growth cycle. For instance, such novel approach has successfully been used to reduce the transfer rate of nitrogen into the environment [101]. Liu et al. [102] also demonstrated that application of control loss fertilizer not only decreases nitrogen runoff and leaching loss by 21.6% and 24.5%, but also augments a 9.8% increase of soil residual mineral nitrogen together with 5.5% increased wheat production as compared to traditional fertilizers. Although, a number of research works were published on this topic, information and research on wider potentiality are still insufficient. Therefore, more research should be carried out to explore novel and promising approaches that can control the migration of other macro- and micro-nutrients as pollutants in micro-scale into the environmental matrix.

## 6. Nanomaterials in Seed Germination, Crop Growth and Quality Enrichment

Nanoscience is a new platform of scientific innovation that involves the development of approaches to a range of inexpensive nanotech applications for enhanced seed germination, plant growth, development and acclimation to environments. The germination of seeds is a sensitive phase in the life cycle of plant, which facilitates seedling development, survival and population dynamics. However, seed germination is largely affected by different parameters including environmental factors, genetic trait, moisture availability and soil fertility [103]. In this concern, an extensive number of studies have shown that the application of nanomaterials has positive effects on germination as well as plant growth and development. For example, the application of multiwalled carbon nanotubes (MWCNTs) positively influences seed germination of different crop species including tomato, corn, soybean, barley, wheat, maize, peanut and garlic (Table 1) [52,53,104,105]. Similarly, nano SiO_2,_ TiO_2_ and Zeolite application positively stimulate seed germination in crop plants [103,106]. Disfani et al. [62] also found that Fe/SiO_2_ nanomaterials have significant potential to improve seed germination in barley and maize. Despite a significant body of research on nanomaterials-induced positive effects on germination, the underlying mechanisms how nanomaterials could stimulate germination still remain unclear. A few studies have demonstrated that nanomaterials have the potential to penetrate the seed coat and enhance the ability of absorption and utilization of water, which stimulates enzymatic system and ultimately improves germination and seedling growth [106,107]. Nonetheless, the mechanism of nanomaterial-induced water uptake inside the seed is still largely unknown. 

In addition to germination, nanomaterials, such as ZnO, TiO_2_, MWCNTs, FeO, ZnFeCu-oxide, and hydroxyfullerenes are reported to increase crop growth and development with quality enhancement in many crop species including peanut, soybean, mungbean, wheat, onion, spinach, tomato, potato and mustard [38,42,89]. For example, carbon nano materials fullerols, as OH-functionalized fullerenes have commonly exerted positive effects on plant growth. Gao et al. [108] demonstrated that fullerenes enhanced hypocotyl growth in *Arabidopsis* by stimulation of cell divisions. It has also been found that seed dressings with Fullerol not only increase fruit number, fruit size, and final yield by up to 128%, but also stimulate the content of bioactive compounds, such as cucurbitacin-B, lycopene, charantin and inulin in fruits of bitter melon (*Momordica charantia*) [109]. Yousefzadeh and Sabaghnia [110] demonstrated that the application of nano-iron fertilizer not only increased the agronomic traits of *Dracocephalum moldavica* with sowing density, but also improved essential oil contents of plants. Similarly, foliar application of nano- zinc and boron fertilizers was found to increase fruit yield and quality, including 4.4–7.6% increases in total soluble solids (TSS), 9.5–29.1% decreases in titratable acidity (TA), 20.6–46.1% increases in maturity index and 0.28–0.62 pH unit increases in juice pH on pomegranate (*Punica granatum*) without affecting any physical fruit characteristics [74]. These findings revealed perspectives of nanomaterials to improve crop yields and product quality. Although the exact mechanism behind promotion of plant growth and enriched quality is not clear, it may be at least partially explained by the potentialities of nanomaterials to absorb more nutrients and water that in turn helps to enhance the vigor of root systems with increased enzymatic activity [38,42]. Moreover, the studies of nutrients on slow/controlled release or control loss of nanofertilizers carried out in water and soil have confirmed that the long term availability of all the doped nutrients to the plant over the full crop period of cultivation is crucial for promoting germination, growth, flowering and fruiting [90]. For example, hydroxyapatite nanomaterial-coated urea fertilizer releases nitrogen slowly and uniformly over up to 60 days, whereas the traditional bulk fertilizer losses only within 30 days with uneven release that reduced the nutrient efficiency of plants and adversely affects crop growth [111]. Conversely, the examination in different studies offers conflicting evidence about the positive nexuses of nanomaterials on seed germination and growth of crops. Such variability may arise due to a number of factors related to nanomaterial characteristics, such as size, shape, surface coating and electronic properties, dose as well as mode of application and the plant species studied [112]. For instance, Zhen et al. [113] demonstrated that the application of TiO_2_ at 2.5% level increased photosynthesis in spinach about 3.13%; however, this decreased beyond 4% of concentration. Disfani et al. [62] also demonstrated that 15 mg kg^−1^ of nano Fe/SiO_2_ increased shoot length of barley and maize seedlings about 8.25% and 20.8%, respectively; however, the shoot length was negatively affected when concentration reached 25 mg kg^−1^, meaning that crop growth depends on the concentration of nanomaterial application. El-Feky et al. [114] showed that plant growth performance was influenced by the mode of nanomaterial application. They found that foliar application of nano Fe_3_O_4_ could significantly enhance total chlorophyll, total carbohydrate, essential oil levels, iron content, plant height, branches/plant, leaves/plant, fresh weight, and dry weight of *Ocimum basilicum* plants compared to that of soil application. 

## 7. Nanomaterials Accelerate Plant Adaptation to Progressive Climate Change Factors

Food security is now a challenging issue for the rising population due to the limited available resources with progressive climate change throughout the world. Progressive climate change refers to the changes in the baseline of climate over time spans, such as temperatures, water deficiency, cold, salinity, alkalinity and environmental pollution with toxic metals. Therefore, the major concern is to enable accelerated adaptation of plants without threatening existing sensitive ecosystems as they strive to cope with environmental stresses [7]. Accomplishing this task requires a multi-pronged strategy, such as activation of plant enzymatic system, hormonal regulation, stress gene expression, regulation of toxic metal uptake and avoiding water deficit stress or flash flood through shortening plant life cycle. Various efforts have been made by researchers to develop technologies and practices towards sustainable agricultural systems by avoiding adverse effects on environmental compartments [38,115]. Advances in nanomaterial engineering suggest that nanofertilizers can increase crop production in existing adverse environments. Salinity stress seriously limits crop production in about 23% of the cultivated lands worldwide [116]. In contrast, it has been reported that application of nano-SiO_2_ improves seed germination, increases plant fresh weight, dry weight and chlorophyll content with proline accumulation in tomato and squash plants under NaCl stress [117,118]. Likewise, Torabian et al. [119] also demonstrated that foliar spray of nano-particles, iron sulfate (FeSO_4_), show positive response to salinity stress tolerance in sunflower cultivars. They reported that nano FeSO_4_ application not only increased leaf area, shoot dry weight, net carbon dioxide (CO_2_) assimilation rate, sub-stomatal CO_2_ concentration (Ci), chlorophyll content, maximum photochemical efficiency of photosystem II (*F*v/*F*m) and iron (Fe) content, but also decreased significant amount of sodium (Na) content in leaves. Recently, it has also been explored that silicon nano-particles (SiNPs) could effectively alleviate UV-B induced stress in wheat [120]. Nano zeolite can improve the long-term availability of nutrients and enhance the germination and growth of plants [103]. Abdel-Aziz et al. [88] showed an excellent effort to explore the application of nanomaterials. They found that the life cycle of nanofertilizer-applied wheat plants was 23.5% shorter (130 days compared with 170 days) for yield production from the date of sowing compared to conventional fertilizer-applied plants. Such an acceleration of plant growth and productivity by the application of nanofertilizers demonstrate their potential as effective tools in agricultural practices, especially in drought-prone, or even in sudden flash flood-prone areas where the early maturity of crops is an important aspect for sustainable crop production. In addition, nanomaterials are found to be effective in detoxification or remediation of harmful pollutants like heavy metals (Table 1). For example, Wang et al. [121] show that the foliar application of nano-Si at 2.5 mM concentration significantly improves Cd stress tolerance in rice plants by regulating Cd accumulation. In another study, the same group showed that nano-Si is also effective against Pb, Cu, and Zn with Cd. It appears that nano-Si fertilizers may putatively have an advantage over traditional fertilizers in reducing heavy metal accumulation [67]. 

Crop productivity is also largely influenced by biotic factors including pests and diseases [122]. To minimize crop losses, farmers have been heavily reliant on pesticides, which adversely affect human health and environmental sustainability. However, recent studies have revealed that nanomaterials could successfully reduce the risks of pests and diseases, thereby minimizing the severity of yield losses and environmental hazards (Table 1). For example, biosynthesized AgNPs obtained using stem extract of cotton plant (*Gossypium hirsutum*) possess a strong antibacterial activity as evidenced by the zone of inhibition for *Xanthomonas axonopodis* pv. *malvacearum* and *Xanthomonas campestris* pv. *campestris*, two major bacterial pathogens of Malvaceae and Brassicaceae family crops, respectively [70]. Metal oxide nanomaterials, such as CuO, ZnO, and MgO could also effectively control many plant and soil borne diseases caused by *Botrytis cinerea, Alternaria alternate, Monilinia fructicola, Colletotrichum gloeosporioides, Fusarium solani, Fusarium oxysporum* fsp Radicis Lycopersici, *Verticillium Dahliae, Phytophthora infestans* and *Ralstonia solanacearum* in many plant species [68,69,71,72]. Hence, the judicious use of nanomaterials can increase crop productivity without disturbing the environmental health. In recent years, researches on the use of nanocomposites have greatly expanded in the field of plant protection due to their high efficacy and eco-friendly nature [123]. For example, Ag-incorporated chitosan nanocomposites (Ag@CS) with fungicides antracol show an increased antifungal activity compared to that of each component alone [124]. Again, the development of nanocomposites of *Bacillus thuringiensis (Bt)* containing active *Bt* has further increased the efficacy and shelf life of pesticides and reduced the dosage required previously. However, the mechanisms behind such actions of *Bt*-based nanocomposites are still to be elucidated [125].

Despite numerous studies on nanomaterials-induced plant growth promotion and stress tolerance, the underlying mechanisms still remain largely uncovered. The influential effects of nanomaterials on crop growth under unfavorable conditions can, at least in part, be explained by the increased activity of enzymatic system [42]. For example, nanomaterials like nano-SiO_2_ or nano-ZnO application increases the accumulation of free proline and amino acids, nutrients and water uptake, and activity of antioxidant enzymes including superoxide dismutase, catalase, peroxidase, nitrate reductase, and glutathione reductase, which ultimately improve plant tolerance to extreme climate events [89,121]. In addition, nanomaterials could also regulate stress gene expression. For example, a microarray analysis showed that a number of genes were up-ragulated or down-regulated by the application of AgNPs in *Arabidopsis* [107]. Among the up-regulated genes, a major part is associated with the response to metals and oxidative stress (cation exchanger, cytochrome P450-dependent oxidase, superoxide dismutase, and peroxidase). In contrast, down regulated genes are related to response to pathogens and hormonal stimuli including systemic acquired resistance, ethylene signaling, and auxin-regulated gene involved in growth and organ size [107]. Such nanomaterials-induced responses are directly involved in plant protection against stresses [116]. However, the response of plants to nanofertilizers varies with the plant species, their growth stages and the nature of nanomaterials used [88]. Therefore, further work is needed to identify the signaling cascades and the genes regulated by specific nanomaterials in different plant species before the technology reaches the farm gate.

## 8. Nanomaterials as Nanosensors: Measurement and Monitoring of Perturbations

Nanomaterial engineering is the cutting-edge research track for sustainable agricultural development. The uses of nanomaterials in precision farming reduce the cost and effort, increase efficiency and lead environmentally sound development. The development of nanosensors to measure and monitor crop growth and soil conditions, nutrient deficiency, toxicity, diseases, and the entry of agrochemicals to the environment would help to assure soil and plant heath, product quality, and overall safety for sustainable agriculture and environmental systems [36]. Naturally, biological organisms have the sense to detect the existing environmental conditions. However, the combination of biology with nanomaterials into sensors has aroused a wider prospect to increase specificity, sensitivity and rapid responses to sense the impairments [38]. For example, nanosensor-based global positioning system (GPS) is used for real time monitoring of cultivated fields throughout the growing season. Such networks of wireless nanosensors monitor the controlled release mechanism via nanoscale carriers employing wireless signals located throughout the cultivated fields. This can assure a real time and comprehensive monitoring of the crop growth and effective high-quality data that provide opportunities for most excellent management practices by avoiding over dose of agricultural inputs [8,126]. The automation of irrigation system by using sensor technology has the great potential to maximize the water use efficiency. In scenario of water limitation, nanosensors estimates soil water tension in real-time coupled with autonomous irrigation control [127]. Likewise, a rapid and accurate detection of insects or pathogens would help in timely application of pesticides or fertilizers to protect the crops from infestation. In this connection, a wireless nanosensor has been developed by Afsharinejad et al. [15] for detecting the insect attack. This sensor distinguishes the emitted volatile organics in many host plant species in relation to the insect types. Sing et al. [128] demonstrated that nano-gold based immunosensor is effective to detect karnal bunt disease in wheat plants. Moreover, in the field of nanobiotechnology, the development of bionic plants by inserting nanoparticles into the cells and chloroplasts of living plants for sensing or imaging objects in their environment and to communicate as infrared devices or even self-powering of plants as light sources has great potentials in precision farming [129,130]. For instance, Giraldo et al. [131] reported that the insertion of single-walled carbon nanotubes (SWNTs) increases electron transfer rate of light-adapted chloroplasts by 49% under in vivo conditions by augmenting photoabsorption. They also showed that SWNTs contributed to the light-harvesting capacity near-infrared fluorescence by suppressing the generation of reactive oxygen species in chloroplast and could influence the sensing process in plants; which resulted in increased photosynthetic efficiency and quantum yield of plants. Therefore, advancements in nanobionic approaches for crops improvement and environmental monitoring open a new window for research into functional plant nanomaterial hybrids [131,132]. 

Plants respond to stress through different physiological changes which are mediated by stress hormones, such as jasmonic acid, methyl jasmonate, and salicylic acid. Fascinatingly, Wang et al. [133] developed a modified gold electrode nanosensor with copper nanoparticles to detect the pathogenic fungus infestation through monitoring the level of salicylic acid in oil seed. Accordingly, multiwalled carbon nanotubes (MWCNTs) are also competent in plant growth study by regulation of hormones, such as auxin which may help scientists to explore how plant roots are acclimatized to their environment, particularly to marginal soils [134]. Ganeshkumar et al. [135] demonstrated that one dimensional potassium niobate (KNbO_3_) nanofibers have great potential to sensing the humidity due to their large surface-volume ratio. The KNbO_3_ nanofiber-based humidity nanosensors display a logarithmic–linear dependence behavior of the conductance with the relative humidity within two second, where the conductance increases from 10^−10^℧ to 10^−6^℧ for a relative humidity varying from 15% to 95% at room temperature. 

Recently, Fang et al. [136] developed a simple, label-free glutathione regulated dual-functional platform based on upconversion nanoparticles (UCNPs)/AuNPs for the turn-on fluorescence detection of acetylcholinesterase (AChE) activity and toxic Cd^2+^ in real water samples. Besides, a significant advancement has been made towards the monitoring and quantification of small amount of pollutants like pesticides. For examples, photosystem II containing biosensors have the potential to bind several groups of pesticides and also can monitor the chemical pollutants. Such nanosensors provide an easy and low-cost effective technology for the detection of specific pesticides with a wide range of organic pollutants before their disposal into the agro-environment [8]. Surely, the intelligent application of nanosensors in agriculture is an emerging tool that provides the assurance of sustainable development by monitoring crop and soil health. However, considering the massive records of research in this area, the convincing applications of nanosensors, especially in field study, are surprisingly insufficient, suggesting a new window for future research.

## 9. Nanomaterials in Pesticide-Based Plant Protection

The assistance of nanotechnology in plant protection products has exponentially increased to achieve higher crop production. In general, conventional crop protection practices involve applications of large-scale and over-dose of fungicides, herbicides and insecticides. Among the applied pesticides, more than 90% are either lost in the environment or unable to reach the target sites essential for effective pest control [43]. This not only increases the expenses of crop production, but also causes the depletion of environmental systems. It is to be noted that the presence of active ingredients in minimum effective concentration of a formulation at the target sites is essential for assurance of better protection of plants from pest invasion and subsequent crop loss. In this connection, the development of new plant protection formulation has long been a very cognitive field of agricultural research. One such technology is nanoformulation or encapsulation of pesticides which has revolutionized the plant protection sector. Nanoformulation of pesticides contains a very tiny number of particles that act as active ingredients of pesticides, whereas other engineered nano-structures also have useful pesticidal properties [137]. The nanoencapsulation of pesticides is the coating of active ingredients of pesticides with another material of various sizes at nano-range where encapsulated materials are referred to as internal phase of the core material (pesticides) and capsulation materials are known as external phase, i.e., the coating nanomaterials [43].

Nanoformulations or encapsulations of pesticides facilitate the persistence or controlled release of active ingredients in root zones or inside plants without compromising effectiveness. On the other hand, conventional formulations of pesticides or herbicides not only limit water solubility of pesticides, but also injure other organisms, leading to increased resistance to target organisms. In contrast, nanoformulations assist to overcome the above-mentioned limitations [1]. For instance, Petosa et al. [138] demonstrated that nanoformulations of pesticides boost crop yields by increasing pesticide efficacy through regulating transport potential of pesticide. They found that nanoformulations combining polymeric nanocapsules and the pyrethroid bifenthrin (nCAP4-BIF) display increased elution with time and enhanced transport potential even upon the addition of fertilizer in loamy sand soil saturated with artificial porewater containing Ca^2+^ and Mg^2+^ cations. This means that nCAP4 could be a promising delivery vehicle of pesticides like pyrethroid in plant protection. This could possibly due to the increased potential of dispersion and wettability of nanoformulations that reduce organic solvent runoff and unwanted pesticidal movement. Moreover, nanomaterials in pesticide formulation show some useful properties like increased stiffness, permeability, thermal stability, solubility, crystallinity and also biodegradability essential for sustainable agro-environmental system [10,137]. More importantly, the timely and controlled release of active ingredients reduce the total amount of pesticides required for pest and disease control, an important feature of integrated pest management (IPM). Furthermore, sustainable agriculture demands minimum use of agrochemicals, so as to protect environmental depletion and others non-target species. Moreover, the minimum use of pesticides reduces the cost involved in crop production. It has been estimated that on a global scale annual agricultural crop losses account for 2000 billion US dollars due to plant diseases, insect pests and weeds, and only in United States for pathogen control efforts simply through fungicide applications cost exceeds $600 million [139,140]. Fascinatingly, in such circumstances, the use of NPs is being reported as efficient alternative to directly suppress pathogen infection and activity, leading to the increased crop growth and yield [141]. For example, halloysites a types of clay nano tubes used as cost-effective carriers of pesticides in agriculture. These nano tubes not only show extended release period of active ingredients (AI), but also provide assurance of better contact with minimum environmental effect [1]. Nano silica is one such example that is hydrophobic in nature and can absorb into the cuticle layer of the insects upon contact, leading to ultimate death of insects [40]. De Jorge et al. [142] showed an excellent effort to explore the importance of nanoformulation in controlled release of AI. They have explored that nanofibers formulation of *Grapholita molesta* (Lepidoptera:Tortricidae) (Busck) pheromone have no effects on mortality over time, suggesting a controlled release of AI and long-term attract-and-kill effect of pheromone and insecticide. 

In addition, many investigations have provided evidence that nanoformulations of pesticides facilitate the widening of plant-based systemic acquired resistance (SAR) against pests. For examples, silica nanosphere formulations can increase ability of pesticides to penetrate through the plant and reach the cell sap, thereby exerting systemic effect to control chewing or sucking type insects like aphid [143]. Such types of hollow formulation also protect pesticides from photodegradation due to direct exposure to sun rays [8]. It has also been observed that nanoformulations alter non-systemic behavior of pesticides [144]. The nonsystemic behavior of ferbam can alter and increase the penetrating potential into tea leaves when formulated with metallic NPs (AuNPs). Such kinds of findings obviously will provide a new frontier of development of pesticide formulations in order to acquire plant-based systemic resistance. However, further study is needed to unravel the behaviors and fate of pesticides and their interactions with biomacromolecules present in living system or environment. Meanwhile, Patil et al. [145] demonstrated that latex fabricated bioactive AuNPs reduced the catalytic potential of trypsin, a vital insect protease and thereby bio-controlling devastating insects. This catalytic inhibition might be due to the interaction of metallic NPs with proteins via covalent interactions, electrostatic interactions or binding to -SH group of amino acid [145].

**Figure 3:**
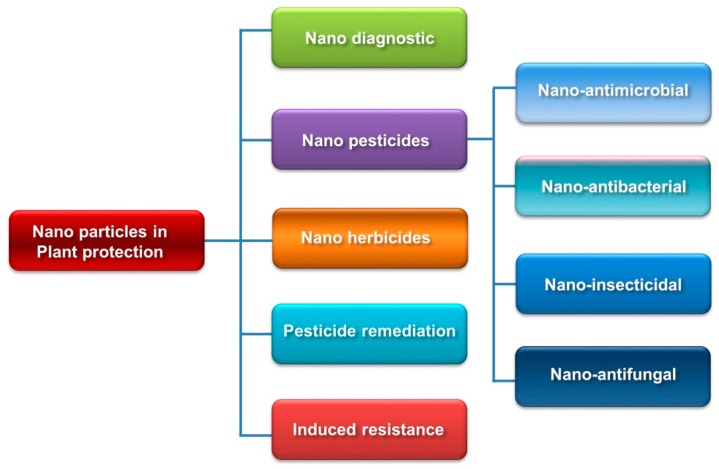
Uses of nanoparticles in plant protection. Nanoparticles can be used for multiple plant protection purposes, such as pathogen detection (nanodiagnostics), pest control (against microbial pathogens, fungi, bacteria and insects), weed control, pesticide remediation, induced resistance and so on. Readers are referred to the text for further details. (Modified and redrawn from references [17,140]).

The potential application of engineered nanomaterials in agriculture is also ascertained in disease and weed management (Figure 3). Inorganic NPs, such as ZnO, Cu, SiO_2_, TiO_2_, CaO, MgO, MnO and AgNPs play important roles in various arena of plant protection including microbial activity and bacterial diseases [141,146]. For example, ZnO nanoparticles have recently been shown to provide effective growth control of *Fusarium graminearum*, *Penicillium expansum*, *Alternaria alternate*, *F. oxysporum*, *Rhizopus stolonifer*, *Mucor plumbeus* and *A. flavus* as well as pathogenic bacteria *Pseudomonas aeruginosa* [1,141,147]. Nano-Cu application was found to be more effective against *Phytophthora infestans* compared to currently available non-nano Cu formulations in tomato [71]. Besides, Si, and TiO_2_ have been found promising to suppress crop diseases directly, through antimicrobial activity. MONPs inhibit the development of fungal conidia and conidiophores which cause ultimate death of fungal hyphae. Likewise, weeds are also considered as serious threat to global agricultural production as they compete with crops for their nutrient, water and light. However, the application engineered nanomaterials containing herbicides provide the solution in eco-friendly manner. For example, Sharifi-Rad et al. [148] demonstrated that germination, root and shoot lengths, fresh and dry weights, and photosynthetic pigments with total protein significantly decreased in weeds exposed to SiO_2_ nanoparticles. Similarly, Kumar et al. [149] showed that herbicide (metsulfuron methyl)-loaded pectin (polysaccharide) nanoparticles are more cytotoxic to *Chenopodium album* plants both in laboratory or in-field conditions and only a very low amount of AI is required compared to the commercial herbicide. Usually, commercial herbicides control or kill the above-ground parts of the weeds without affecting the below-ground parts like rhizomes or tubers. As a result, the regrowth of weeds occurs; however, nanoherbicides prevent the regrowth of weeds [1]. Thus, the nanomaterials in pesticides, fungicides and herbicides have a tremendous scope in sustainable agricultural development.

## 10. Conclusions

**Figure 4:**
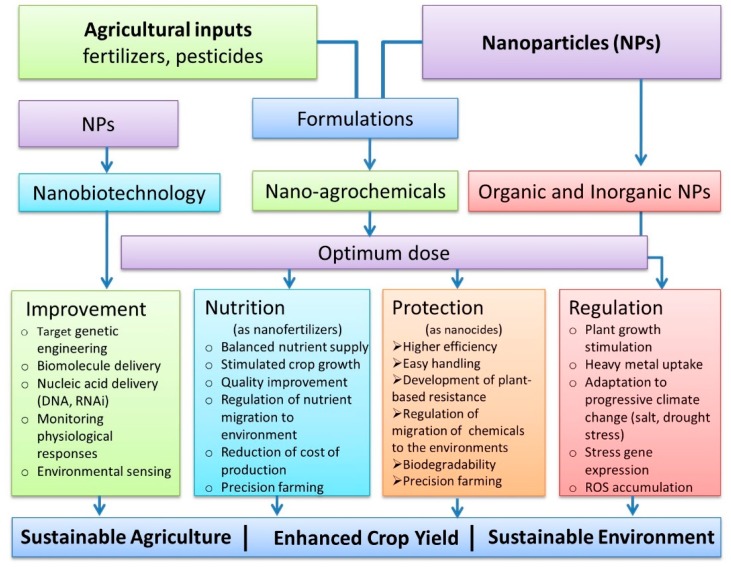
Simplified overview of potential applications of nanomaterials in sustainable agriculture production. Improvement of crop productivity using nanomaterials in target crop genetic engineering and smart monitoring of plant response to environments with nanosensors. Applications of nanomaterials to increase crop productivity using nanofertilizers and nanopesticides. Improvement of plant growth and adaptation to progressive climate changes using nanomaterials.

In the field of agriculture, nanotechnology has been used to heighten the crop production with quality enrichment by improving farming systems as schematically shown in Figure 4. The emergence of engineered nanomaterials and their actions within the frame of sustainable agriculture have revolutionized world agriculture canvass dramatically by novelty, fast growth and enormity to meet the projection of global food demand. In sustainable agriculture, the protection of the environment from pollution is the crucial target for trade, and nanomaterials provide an assurance of better management and conservation of inputs to plant production. The potential of nanomaterials encourages a new green revolution with reduced farming risks. However, there are still huge gaps in our knowledge of the uptake capacity, permissible limit and the ecotoxicity of different nanomaterials [9,150]. Therefore, further research is urgently needed to unravel the behavior and fate of altered agriculture inputs and their interaction with biomacromolecules present in living systems and environments.
